# Supplementary figures and images for: Viral Epitope Scanning Reveals Correlation between Seasonal HCoVs and SARS-CoV-2 Antibody Responses among Cancer and Non-Cancer Patients
Source: Viruses. 2024 Mar 13;16(3):448. doi: 10.3390/v16030448 (PMC10975915; doi:10.3390/v16030448)

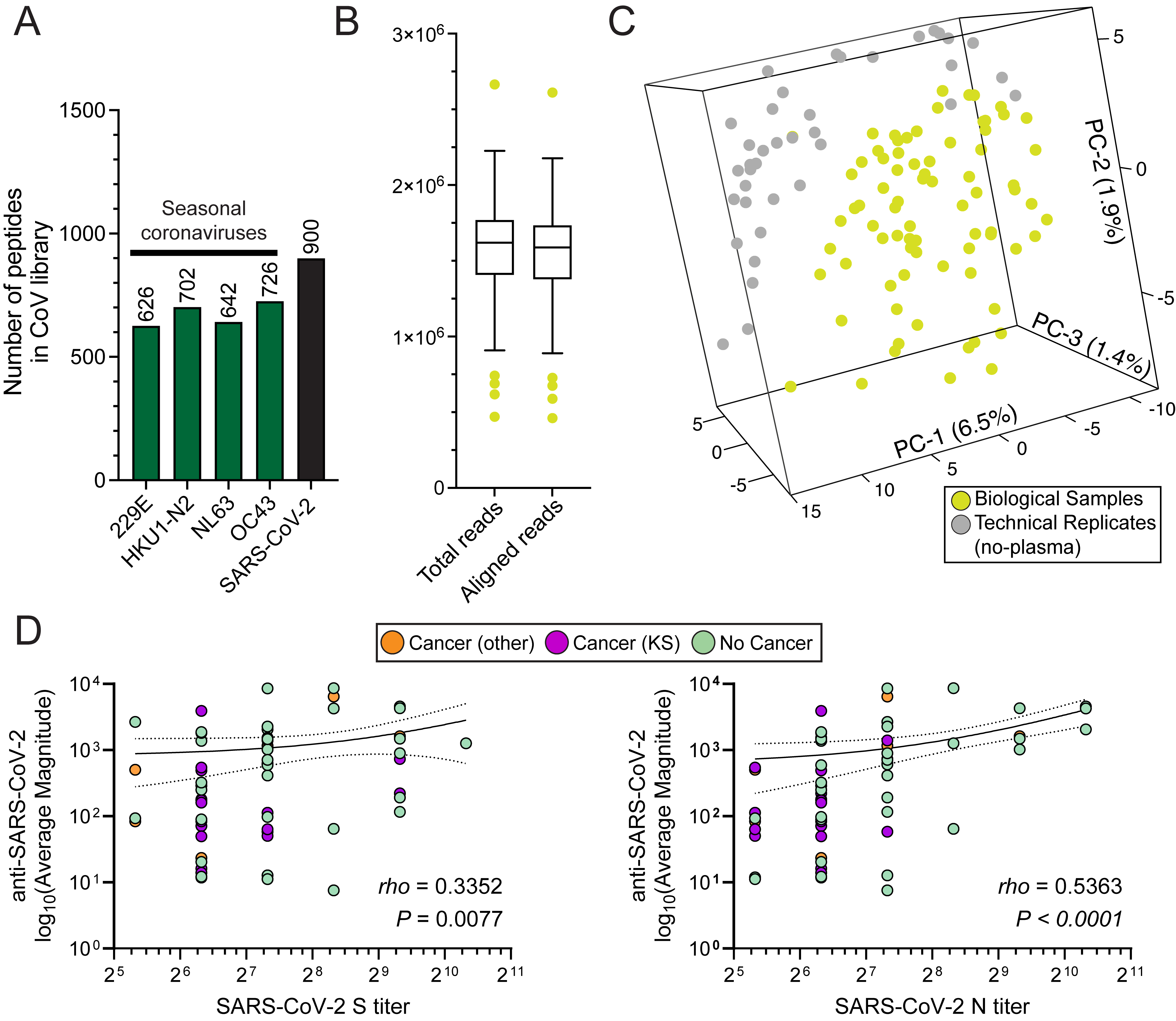

Supplement: Supplementary file 1 [file viruses-16-00448-s001.zip › viruses-2869637-supplementary.tif]
